# Investigation of Vaping Fluids Recovered From New York State E-Cigarette or Vaping Product Use-Associated Lung Injury Patients

**DOI:** 10.3389/fchem.2021.748935

**Published:** 2021-10-27

**Authors:** Shijun (Jimmy) Lu, Lingyun Li, Bryan C. Duffy, Mark A. Dittmar, Lorie A. Durocher, Deepika Panawennage, Em R. Delaney-Baldwin, David C. Spink

**Affiliations:** ^1^ Laboratory of Organic Analytical Chemistry, Wadsworth Center, New York State Department of Health, Albany, NY, United States; ^2^ Department of Environmental Health Sciences, School of Public Health, University at Albany, State University of New York, Albany, NY, United States

**Keywords:** electronic cigarettes, vaping fluid, EVALI, cannabis, vitamin E acetate, diluents, terpenes, pesticides

## Abstract

E-cigarette or vaping product use-associated lung injury (EVALI) is a serious pulmonary condition that is associated with the extended use of certain vaping products. EVALI was first characterized in the summer of 2019 and has since been reported in all 50 U.S. states. From August 2019 through June 2021, the New York State Department of Health has reported more than 197 confirmed cases emanating from all regions of the state. The Wadsworth Center at the New York State Department of Heath received vaping cartridges recovered from EVALI patients for chemical analysis of their contents. Untargeted analytical methods using gas chromatography-mass spectrometry and liquid chromatography-high-resolution mass spectrometry as well as targeted analyses for a variety of analytes including cannabinoids, pesticides, vitamin E acetate (VEA) and mycotoxins were used to characterize the composition of the vaping fluids and several commercial vaping fluid additives. From the analyses of the 284 e-cigarette devices recovered from patients, 82 were found to be nicotine-containing pods, and 202 devices containing cannabis oil, apparently from unauthorized or black-market dealers. The fluids from the cannabis-oil cartridges tended to have lower levels of THCs (Δ^9^-tetrahydrocannabinol + Δ^8^-tetrahydrocannabinol) and total cannabinoids compared with those of commercially produced formulations and contained significant levels of diluents including VEA, medium-chain triglycerides, polyethylene glycol, and castor oil. VEA was the diluent most frequently detected, which was present in 132 (65.3%) of the vaping fluids that contained cannabis oil. When present, VEA ranged from 2.0 to 67.8% of the total mass of the oil with a mean content of 37.0%. In some cases, two or three diluents were detected in the same sample. The ratio of VEA to THCs varied widely, from 0.07 to 5.34. VEA and specifically the high ratios of VEA to THCs in black-market vaping fluids may be causative in EVALI. The safety of additional components and additives that are present in vaping fluids are likewise of concern.

## Introduction

Coincident with the development of the e-cigarette, vaping has become a popular way to use in both nicotine and cannabis products (Gaub et al., 2019; Kumar et al., 2019; DiSilvio et al., 2021). First introduced into the US in 2006, e-cigarettes consist of a wicking material that draws the vaping fluid from a reservoir to a battery-powered metal coil that, when heated, vaporizes the fluid (Brown and Cheng 2014; Chun et al., 2017). The composition of vaping fluids varies, but they generally contain either an aqueous-based nicotine solution or a cannabis oil. E-cigarettes have been marketed as a safer alternative to traditional smoking, as the number of harmful byproducts from combustion of tobacco is greatly reduced (Giroud et al., 2015; Margham et al., 2016). However, the health effects of vaping various oils, diluents, and flavoring agents that are present in various devices are not fully understood.

Despite numerous rescheduling attempts, marijuana, or cannabis, has remained a U.S. Drug Enforcement Agency Schedule I controlled substance. As a result, cannabis vaping fluids/devices have until recently been illegal. Changing regulations now permit medical and/or adult recreational use in 47 U.S. states; however, numerous restrictions regarding cannabis use remain (NCSL 2021), and black markets for cannabis products persist, including those for illicit vaping devices. The composition of these black-market vaping fluids is a major concern, since regulators cannot provide oversight of the manufacturing practices and the additives used. Consumers may thus be exposed to significant health risks when using illicit vaping products.

The medical condition that is now known as E-cigarette or vaping product use-associated lung injury (EVALI) was first reported in June of 2019 in the U.S. states of Illinois and Wisconsin. This initial outbreak involved 98 patients, mostly young (median age 21 years) and male (79%), who presented with bilateral infiltrates upon chest imaging and had respiratory, gastrointestinal, and constitutional symptoms (Layden et al., 2020). These patients were suspected of having a malady that was not caused by an infectious agent, but rather a condition caused by a chemical component arising from the vaping fluid. As of February 18, 2020, a total of 2,807 hospitalized EVALI cases and 68 deaths had been reported to the U.S. Centers for Disease Control and Prevention (CDC) from all 50 U.S. states, the District of Columbia and the U.S. territories of Puerto Rico and the U.S. Virgin Islands (CDC 2020). The widespread occurrence of the condition spurred numerous investigations into the possible cause(s) of EVALI.

In August of 2019, the Wadsworth Center of the New York State Department of Health (NYSDOH) began receiving vaping devices associated with EVALI cases that were submitted from poison control centers and health care providers in New York State for chemical analysis. During the early investigation of EVALI, it was discovered that many of the cannabis vaping fluids that were associated with EVALI cases contained high levels of vitamin E acetate (VEA). In light of these findings, NYSDOH issued a health advisory and reported the presence of VEA in illicit vaping cartridges recovered from EVALI patients (NYSDOH 2019; Duffy et al., 2020). VEA has since been strongly linked with the etiology of EVALI, as chemical analysis showed the presence of VEA in the vast majority of bronchoalveolar lavage (BAL) samples from the of EVALI patients (Blount et al., 2019; 2020). Potential mechanisms of toxicity of VEA that may be relevant to EVALI have been identified (Wu and O'Shea, 2020; Jiang et al., 2020; Muthumalage et al., 2020).

Our previous report described the findings from the analysis of samples associated with the first 10 EVALI cases in New York State for which vaping products were available (Duffy et al., 2020). The current paper reports on a detailed analysis of the vaping fluid compositions of 284 samples from 83 EVALI patients received at the Wadsworth Center from August of 2019 through June of 2021. Both cannabinoid- and nicotine-containing products were analyzed. Our results show a variety of important analytical findings on diluents and combinations thereof in vaping fluids and the contamination of the fluids with pesticide residues. We report that VEA-containing cannabis vaping fluids associated with recent cases of EVALI in New York continue to be received and analyzed by our laboratory as of June of 2021.

## Materials and Methods

### EVALI Case Identification and Sample Collection

EVALI cases reported to the NYSDOH by health care providers, and medical records provided were reviewed by medical staff of the Center for Environmental Health at the NYSDOH. EVALI diagnoses were based on criteria and case definitions established by the CDC (CDC 2019). Vaping devices provided by the patients or their guardians that were determined to be associated with EVALI cases were submitted to the Wadsworth Center for analysis after referral from poison control centers in New York State. The samples received were generally either cannabinoid-containing vaping cartridges or nicotine-containing pods. A single device or as many as 23 devices were received in association with a single EVALI case. For cannabis vaping cartridges, the units were disassembled, and a sample of the cannabis oil was recovered with the tip of a spatula. When the cartridge appeared to be empty, the reservoir section of the device was placed in a centrifuge tube and centrifuged at 2,000 x *g* for 2 min. This procedure often produced enough material for analysis. A portion of the viscous cannabis oil, about 10 mg or whatever was recovered, was weighed to ± 0.01 mg and dissolved in 50:50 methanol:acetonitrile to give a sample concentration of 10 mg/ml that was further diluted as appropriate for a suite of analyses. The fluids from nicotine pods were recovered using a micropipette. The recovered portion was likewise weighed to ±0.01 mg and dissolved in 50:50 methanol:acetonitrile for further analyses.

### Chemicals and Standards

The following certified cannabinoid reference standards were purchased from Cerilliant (Round Rock, TX, United States): cannabidiolic acid (CBDA), cannabigerolic acid (CBGA), cannabigerol (CBG), cannabidiol (CBD), tetrahydrocannabivarin (THCV), cannabinol (CBN), Δ^9^-tetrahydrocannabinol (Δ^9^-THC), Δ^8^-tetrahydrocannabinol (Δ^8^-THC), cannabichromene (CBC), cannabidivarin (CBDV), and Δ^9^-tetrahydrocannabinolic acid-A (THCA). Primary analytical standards and 13C-isotopically labeled internal standards for aflatoxins B1, B2, G1, G2, and ochratoxin A were purchased from Romer Labs (Union, MO, United States). VEA and vitamin E-*d*_6_ ((±)-α-tocopherol-*d*_6_) were from Cerilliant (Round Rock, TX, United States). The myclobutanil analytical standard was purchased from Accustandards (New Haven, CT, United States). Polyethylene glycol (PEG), USP-grade castor oil, norgestrel, and myclobutanil-(phenyl-*d*_4_) was purchased from MilliporeSigma (St. Louis, MO, United States). Myglol, a medium-chain triglyceride (MCT) oil, was from Warner Graham (Cockeysville, MD, United States). A Piperonyl butoxide (PBO) analytical standard was purchased from Agilent Technologies (Santa Clara, CA, United States), and the corresponding piperonyl butoxide-*d*_9_ (PBO-*d*_9_) internal standard was from Toronto Research Chemicals (Toronto, Canada). Mixtures containing 884 pesticide and pesticide metabolite standards for use in pesticide screening were provided by Dr. Jon Wong of the Center for Food Safety and Applied Nutrition, US Food and Drug Administration (FDA), College Park, MD, United States. Ammonium formate, formic acid, methanol, acetonitrile, and water were HPLC-grade. All other reagents used were analytical grade.

### Analytical Methods

#### Untargeted Analysis

Untargeted analyses were performed using both gas chromatography-mass spectrometry (GC-MS) and liquid chromatography-high-resolution tandem mass spectrometry (LC-HRMS/MS). For GC-MS analysis, the initial extracts were injected onto a GC-MS system comprised of a model 6890A GC interfaced with a model 5973N quadrupole mass selective detector (Agilent). Compounds were resolved on a DB5-MS column (60 m × 250 µm ID; 0.25 µm film thickness; Agilent J&W) with helium as the carrier gas at 1.5 ml/min. The MS transfer line and ion source were at 300°C and 235°C, respectively. The initial oven temperature was 90°C for 1min, followed by a ramp of 2°C/min to 320°C and a hold at 320°C for 25 min. After a solvent delay of 7 min, full-scan mass spectra were recorded over the 50–550 m*/z* range in the electron ionization mode. For compound identification, data were queried against the National Institute of Science and Technology (NIST) Mass Spectral Library 11, the latest Cayman toxicology mass spectral library, and an in-house built mass spectral library.

For untargeted analysis using LC-HRMS/MS, a system comprised of a Shimadzu HPLC interfaced with a SCIEX TripleTOF 6,600 mass spectrometer was employed as described (Duffy et al., 2020). Briefly, a Poroshell EC-C18 HPLC column (Agilent, 2.1 × 100 mm; 2.7 µm particle size) was used for analyte separations. Gradient elution was performed with mobile phases A (0.1% v/v formic acid in water) and B (5 mM ammonium formate in methanol). The mass spectrometer was operated in the positive-ion ESI mode for high-resolution MS and MS/MS acquisition. High-resolution MS and MS/MS spectra were recorded using the information-dependent acquisition technique. Data were acquired using Analyst Software (SCIEX, version 1.6.1) and data were processed using PeakView software (SCIEX, version 2.1). An accurate-mass compound library that was prepared in-house as well as other commercial and public domain databases that included data for synthetic cannabinoids, opiates, synthetic opioids, stimulants, numerous drugs of abuse and previously identified cannabis oil diluents and additives were used for compound identification.

#### Targeted Analysis

Quantitation of cannabinoids was performed using HPLC with photodiode array detection (NYSDOH 2018b; Li et al., 2019). This method has been certified for use in the New York State Medical Marijuana Program (NYSMMP) by the New York State Environmental Laboratory Approval Program (ELAP) according to TNI standards and has been used in the NYSMMP since 2015 for the analysis of thousands of NYSMMP samples. The method employs a Shimadzu (Kyoto, Japan) HPLC system with an SPD-M20A photodiode array detector. Cannabinoids (CBDA, CBGA, CBG, CBD, THCV, CBN, Δ^9^-THC, Δ^8^-THC, CBC, CBDV, and THCA) are resolved on an Agilent Poroshell 120 column (3.0 × 150 mm with 2.7 µm particle size) using isocratic elution at 73% v/v acetonitrile in water with 0.1% v/v formic acid and quantitation of absorbance at 227 nm relative to that of the norgestrel internal standard. Six-point calibration curves for each cannabinoid over the range of 0.19–45.0 μg/ml, plotting area ratios of the absorbance at 227 nm for the analytes to that of the internal standard against analyte concentration. The limit of detection (LOD) for each analyte was determined at the 99% confidence level from the analysis of seven blank samples that were fortified with low levels of each cannabinoid. The limit of quantitation (LOQ) for each cannabinoid was defined as five times the LOD, provided that this value was not below the lowest concentration calibrant of the calibration curve.

The analysis of mycotoxins was performed using LC-MS/MS with an ELAP-accredited method developed by NYSDOH Medical Marijuana Laboratory (NYSDOH 2018a) for the quantitation of aflatoxins B1, B2, G1, G2, and ochratoxin A in medical marijuana products, modified for use with limited amounts of sample. Quantitative analysis of the fungicide myclobutanil and the insecticide synergist PBO was conducted using a method developed and certified by the NYSDOH Medical Marijuana Laboratory using LC-MS/MS*.*

The quantitative analysis of VEA in vaping fluids was performed using GC-MS with electron ionization and operation in the selected-ion monitoring mode with vitamin E-*d*_6_ as the internal standard (Duffy et al., 2020). The analytical system used was composed of a model 7890B GC with model G4513A autosampler interfaced with a 5977A MSD and Mass Hunter Version B07.01 SP/Build 7.1.524.1 software (Agilent Technologies). An Agilent HP-5MS column (30 m × 250 µm with 0.25 µm film thickness) was used with the following temperature program: an initial temperature of 90°C for 1 min followed by an increase at 8°C per min to a final temperature of 290°C, a hold for 4 min, an increase at 10°C per min to 300°C, and a hold for 1 min. Ions of *m/z* 430 and 165 were monitored for VEA; *m/z* 436 and 171 were monitored for the vitamin E-*d*_6_ internal standard. Dwell times were 50 ms. A calibration range of 0.039–2.5 μg/ml was established for VEA. The original vaping fluid solutions at 10 mg/ml typically required an additional dilution of 1,000- to 10,000-fold for analysis.

### Screening for Pesticides and Pesticide Metabolites

A non-targeted data acquisition for target analysis technique using ultra high-performance liquid chromatography (UHPLC) coupled with a quadrupole-orbitrap mass spectrometer (QE-Orbitrap-MS) that is based on previous studies of screening for pesticide residues was used (Wang et al., 2019). The instrumental system used was a Vanquish UHPLC with a Hypersil GOLD column (100 × 2.1 mm with 1.9 µm particle size, Thermo Fisher Scientific, Waltham, MA, United States) interfaced with a high-resolution QE-Orbitrap-MS (Thermo Fisher Scientific) operating in the positive-ion ESI mode (Duffy et al., 2020). A pesticide database that was kindly provided by Dr. Jon Wong of the Center for Food Safety and Applied Nutrition, U.S. FDA, College Park, MD allowed identification of pesticide residues.

## Results and Discussion

### Reported Cases of EVALI in New York State

**FIGURE 1:**
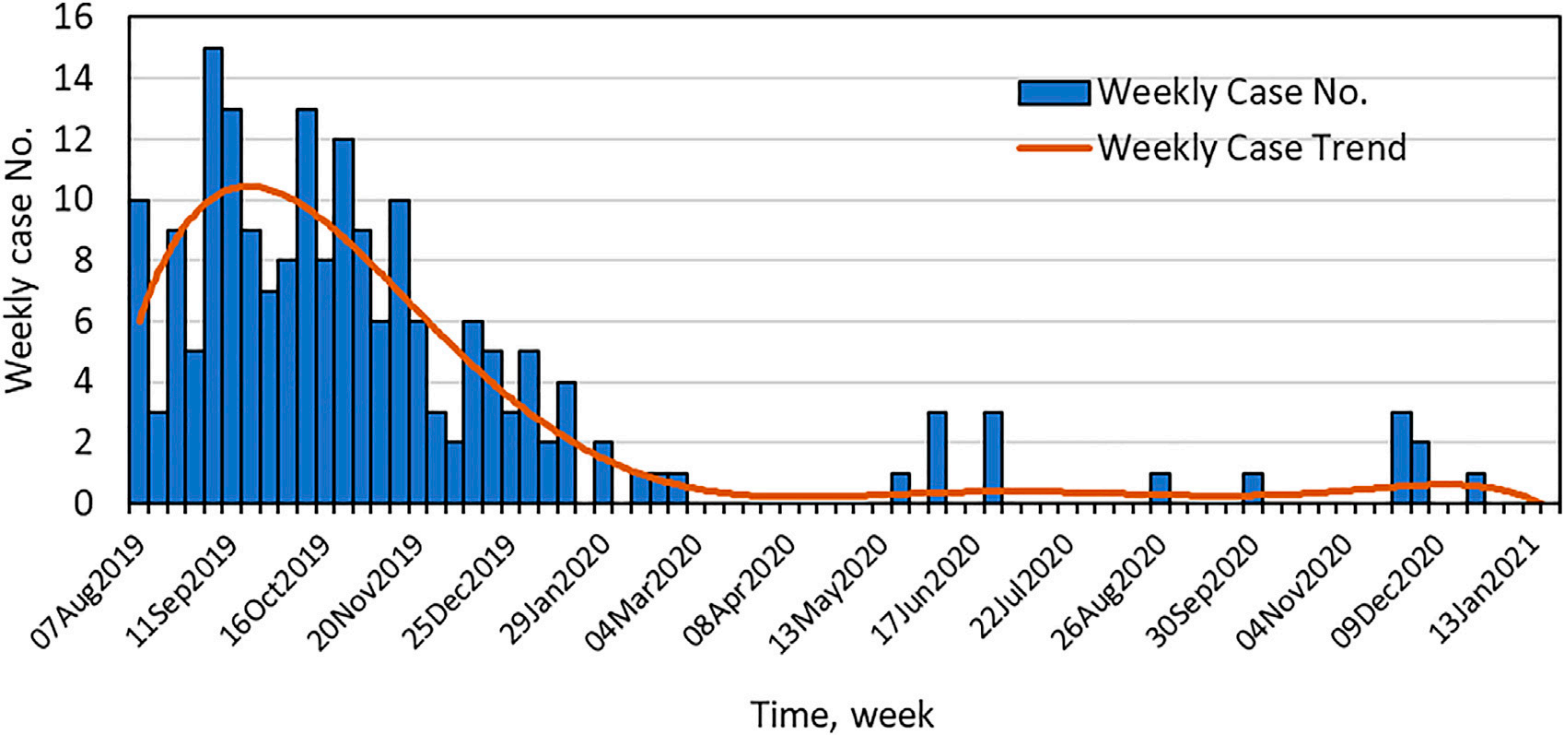
Time course of the EVALI outbreak in New York State. Shown are the number of EVALI cases reported to the NYSDOH per week and the weekly case trend.

As in many parts of the U.S., cases of EVALI first appeared in New York State in early August of 2019. The weekly number of EVALI cases reported to the NYSDOH are shown in Figure 1. The incidence of reported EVALI cases peaked in mid to late September of 2019, then rapidly declined to the end of 2019, stabilizing at 1 to 3 cases or less per week by mid-January 2020. The decline in EVALI cases in New York State came soon after a press release by NYSDOH on Sept. 5th, 2019, warning against the use of black-market vaping products and announcing VEA as the focus of the investigation of lung injury associated with vaping (NYSDOH, 2019), and the preliminary report by Layden and others (Layden et al., 2020) published on-line Sept. 6th, 2019 relating pulmonary illness to e-cigarette use in Illinois and Wisconsin. A few additional EVALI cases were reported later 2020 and sporadically through mid-2021.

### Characterization of EVALI Vaping Fluids–Major Components

Untargeted chemical analyses were performed on a total of 284 vaping products obtained from 83 patients. Results from the GC-MS and LC-HRMS/MS analyses confirmed the sample types as cannabinoid-containing or nicotine-containing and served to identify the major components.

When separated according to cases, the most prevalent association of EVALI was with cannabinoid-containing vaping fluids (Table 1). For many patients, only cannabinoid-containing vaping devices were received, while both cannabinoid-containing and nicotine-containing devices were obtained from others. In 24% of the patients, only nicotine-containing products were received. A summary of the cannabinoid profiles found in the cannabis vaping products is presented in Table 2. Of cannabis vaping products obtained, 194 (96%) contained Δ^9^-THC above the LOQ of 1% by mass. The Δ^8^-THC isomeric form was also found in 28 of the samples at varying levels. The Δ^8^-THC-containing samples did not appear to represent a homogenous subgroup of samples. Varying amounts of CBG, CBN, CBD, THCA, CBC and THCV were quantified in the cannabis vaping fluids. One of the vaping fluids that contained nicotine also contained a low level of CBD. This sample showed phase separation, which would be expected for such a sample, as cannabis oil is lipophilic in nature and is immiscible with aqueous-based solutions of nicotine and nicotine salts.

**TABLE 1:** Types of samples associated with EVALI cases.

Sample types submitted^a^	Number of cases (%)
Cannabinoids only	39 (47.0%)
Both cannabinoids and nicotine	24 (28.9%)
Nicotine only	20 (24.1%)
Total	83 (100.0%)

a Untargeted screening was performed on a total of 284 vaping products from 83 patients.

**TABLE 2:** Cannabinoids in vaping fluids.

Cannabinoid	N (%)^a^	Min %^b^	Max %^c^	Mean ± S.D.
CBC	11 (5.4%)	1.2	2.6	1.7 ± 0.4
CBD	25 (12.4%)	0.8	44.9	7.2 ± 9.5
CBG	102 (50.5%)	1.2	5.3	2.0 ± 0.8
CBN	86 (45.6%)	1.1	16.9	3.2 ± 2.3
Δ^8^-THC	29 (14.4%)	1.9	88.8	26.1 ± 16.6
Δ^9^-THC	194 (96.0%)	1.1	88.7	33.1 ± 18.9
THCA	17 (8.4%)	1.9	87.9	11.8 ± 22.8
THCV	1 (<1.0%)	2.3	2.3	2.3
CBDA	1 (<1.0%)	4.0	4.0	4.0
CBDV	N.D^d^	N/A^e^	N/A	N/A
CBGA	N.D	N/A	N/A	N/A

a Number of samples containing the cannabinoid and as a percentage of the total cannabinoid-containing samples.

b Minimum concentration observed in mass%.

c Maximum concentration observed in mass%.

d N.D, not detected in any sample (below the reporting limit of 1.2% by mass).

e N/A, not applicable.

### Identification of Diluents in Cannabis Vaping Fluids

**FIGURE 2:**
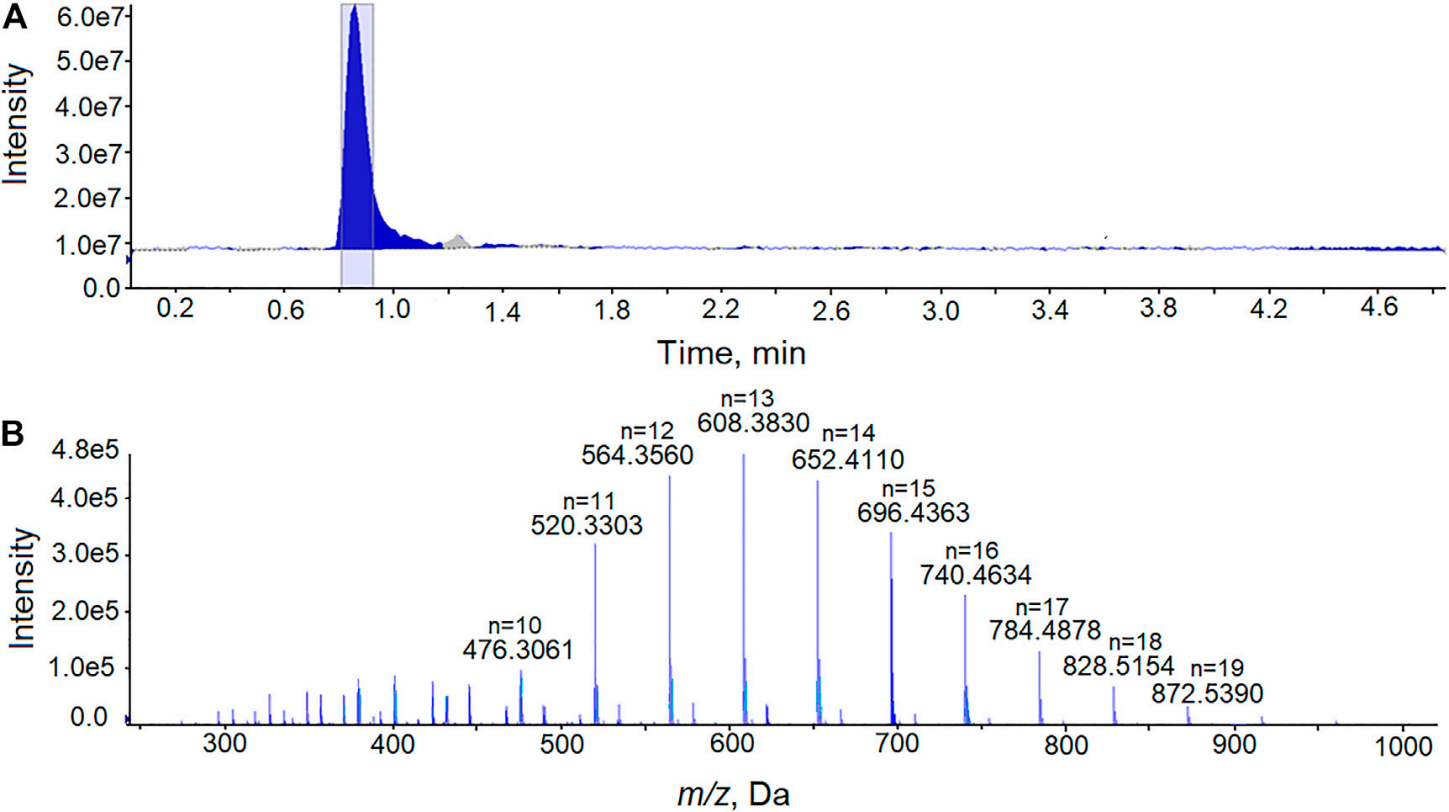
Analysis of PEG in a cannabis vaping fluid using LC-HRMS. **(A)** The total ion current chromatogram from the analysis of an extract of a cannabis vaping fluid. **(B)** Mass spectrum recorded at retention time 0.81–0.92 min showing the [(C_2n_H_4n+2_O_n+1_)+ NH_4_]^+^ ions of PEG with the peaks for polymers of n = 10 through n = 19 denoted.

All 202 cannabis vaping products appeared to be illicit or black-market products since none had packaging or markings indicative of products approved by the NYSMMP. Diluents present in vaping fluids that were not approved by the NYSMMP were identified in 185 (92%) of the vaping fluids. VEA, MCT and PEG were repeatedly identified as diluents in the EVALI-associated vaping products. The identification of PEG in a vaping fluid is shown in Figure 2. PEG of a polymer of the formula H-(OCH_2_CH_2_)_n_-OH, in which n can vary from less than ten to several thousand. The PEG polymers identified as vaping fluid diluents were typically of average molecular mass 600. In positive-ion ESI-MS in the presence of ammonium acetate, PEG is detected as a series of peaks with the formula [(C_2n_H_4n+2_O_n+1_) + NH_4_]^+^. In Figure 2, the peaks corresponding to PEG polymers with n = 10 through n = 19 are denoted. In each case, the *m/z* assignments are within 6 ppm of the theoretical values for the ammonium ion adducts of the PEG polymers.

**FIGURE 3:**
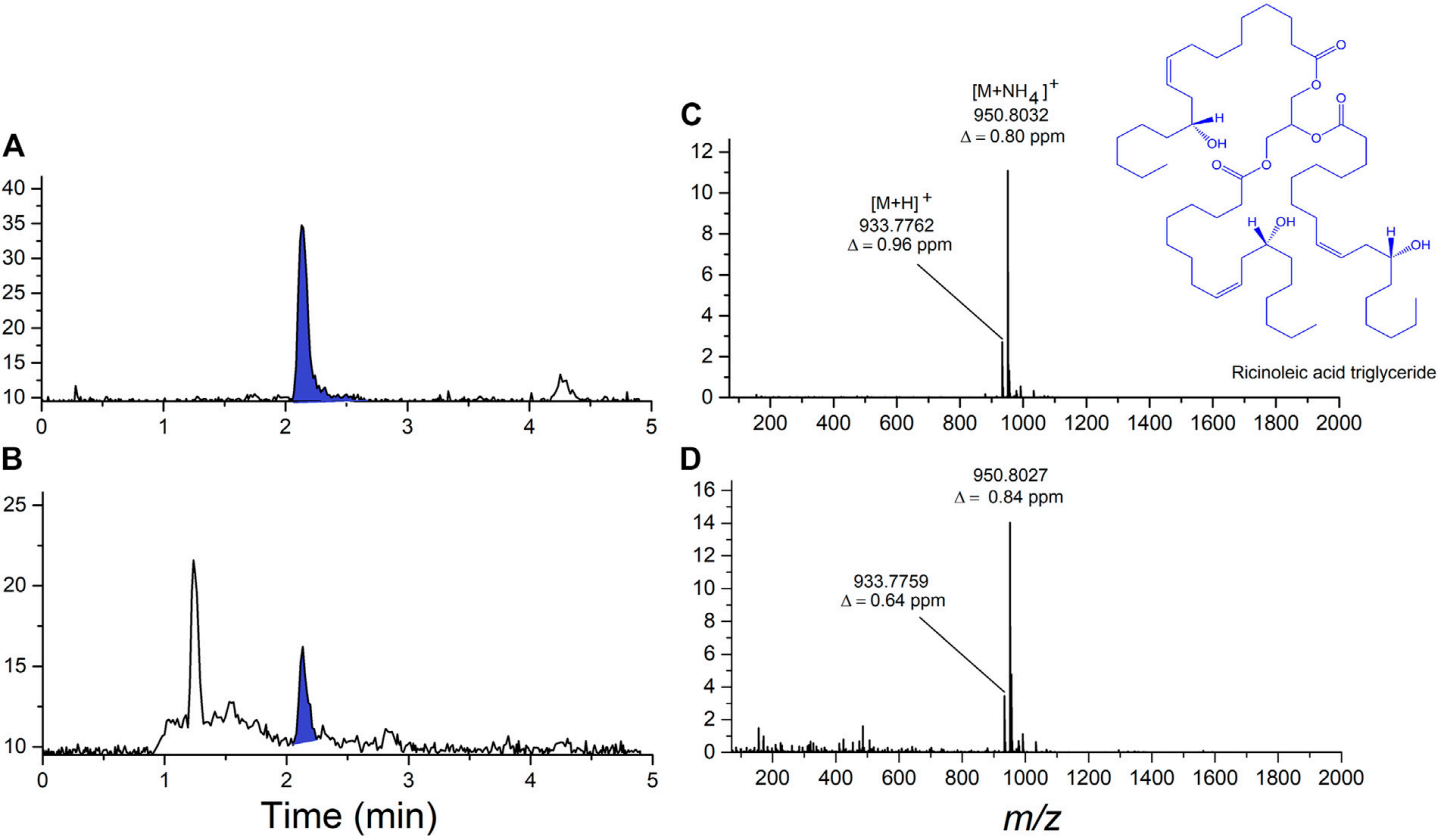
Analysis of ricinoleic acid triglyceride, the major component of castor oil, in vaping fluid using LC-HRMS. **(A)** Total ion current chromatogram from the analysis of USP-grade castor oil standard showing the ricinoleic acid triglyceride peak shaded in blue. **(B)** Total ion current chromatogram from the analysis of an extract of a cannabinoid-containing vaping fluid. **(C)** High-resolution ESI mass spectrum of ricinoleic acid triglyceride from the castor oil standard. **(D)** High-resolution ESI mass spectrum of ricinoleic acid triglyceride present in the cannabinoid-containing vaping fluid.

In the initial untargeted analysis using LC-HRMS/MS, two of the vaping fluid samples showed the same prominent unknown component. Interpretation of accurate-mass data lead to the hypothesis that the unknow diluent was castor oil. The major component of castor oil is ricinolein, or ricinoleic acid triglyceride. Analysis of a vaping fluid sample together with a USP-grade castor oil standard and are shown in Figure 3. Ricinoleic acid triglyceride was confirmed as the major component of castor oil, eluting form the column with a retention time of 2.05 min (Figures 3A,B). Upon electrospray ionization in the presence of ammonium acetate, ricinoleic acid triglyceride produces a dominant [M + NH_4_]^+^ ion that was 4-fold more intense than the [M + H]^+^ (Figures 3C,D). The accurate-mass measurements were consistent with the component in the vaping fluids being ricinoleic acid triglyceride, as were MS/MS spectra obtained from the [M + H]^+^ and [M + NH_4_]^+^ ions as precursors.

VEA was the diluent most frequently detected in the cannabis vaping samples (Table 3). For a majority of the EVALI patients (76%), cannabis vaping fluids were associated with the condition. For EVALI cases in which cannabis vaping products were submitted to the laboratory for analysis, 84% of the time at least one VEA-containing fluid was among the samples received among the patients vaping products. MCT, PEG and castor oil were also detected as diluents in the vaping fluids, although less frequently than VEA. Some products were found to contain two or three diluents. Binary combinations of VEA, MCT and PEG were observed, as were samples in which all three of these diluents were present in combination (Table 3).

**TABLE 3:** Diluents found in cannabis vaping fluids submitted to NYSDOH.

Diluent	No. of samples	No. of associated cases^a^
No diluents	17	12
VEA only	107	51
MCT only	42	18
PEG only	8	4
Castor oil only	2	2
VEA + MCT	19	8
VEA + PEG	4	3
MCT + PEG	1	1
VEA + MCT + PEG	2	1

a The number of cases in which at least one sample has the indicated diluent profile.

**FIGURE 4:**
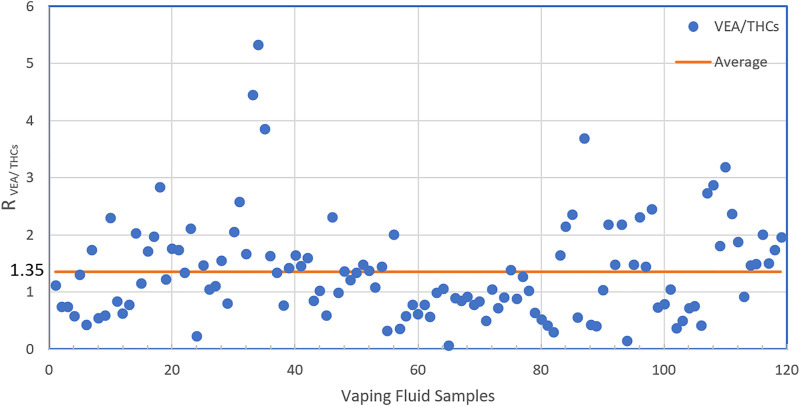
The Ratio of VEA to THCs in illicit vaping fluids. In the vaping samples found to contain VEA, the ratios of VEA to THCs (Δ^9^-THC + Δ^8^-THC) by mass in the individual samples are shown.

When diluents are used in the black-market cannabis industry, the supply of expensive cannabis oil can be extended, and profits can be maximized. VEA must have seemed to be a nearly ideal diluent for black-market cannabis vapor fluids, as it is nearly tasteless, odorless, and has very similar viscosity and color to undiluted cannabis oil, even when mixed at high ratios with cannabis extract. The vaping fluids from illicit vaping products generally had low cannabinoid content (on average, ∼30% THC) and often contained as much or more VEA than total cannabinoids. The ratio of VEA to THCs varied widely, from 0.07 to 5.33 with an average of 1.35 (Figure 4). In the vaping fluids that contained VEA, the mean ± SD VEA concentration was 37.0 ± 15.4 mass%, and the range of values was 2.0–67.8. Since cannabis product users tend to self-titrate their dose according to the response they obtain and their tolerance (Barnes, 2006), as the ratio of VEA to THC in the fluid increases, cannabis users will inhale more vaping fluid, and thus more VEA, to achieve the same dose of THC. As noted, MCT and PEG were present in some of the VEA-containing fluids as additional diluents. These results are in sharp contrast to the archetypical vaping fluids analyzed for the NYSMMP, which have very high cannabinoid content, 80–90% by mass, and are excipient-free, *i.e*., they do not contain diluents.

### Contaminants in Vaping Fluids

**FIGURE 5:**
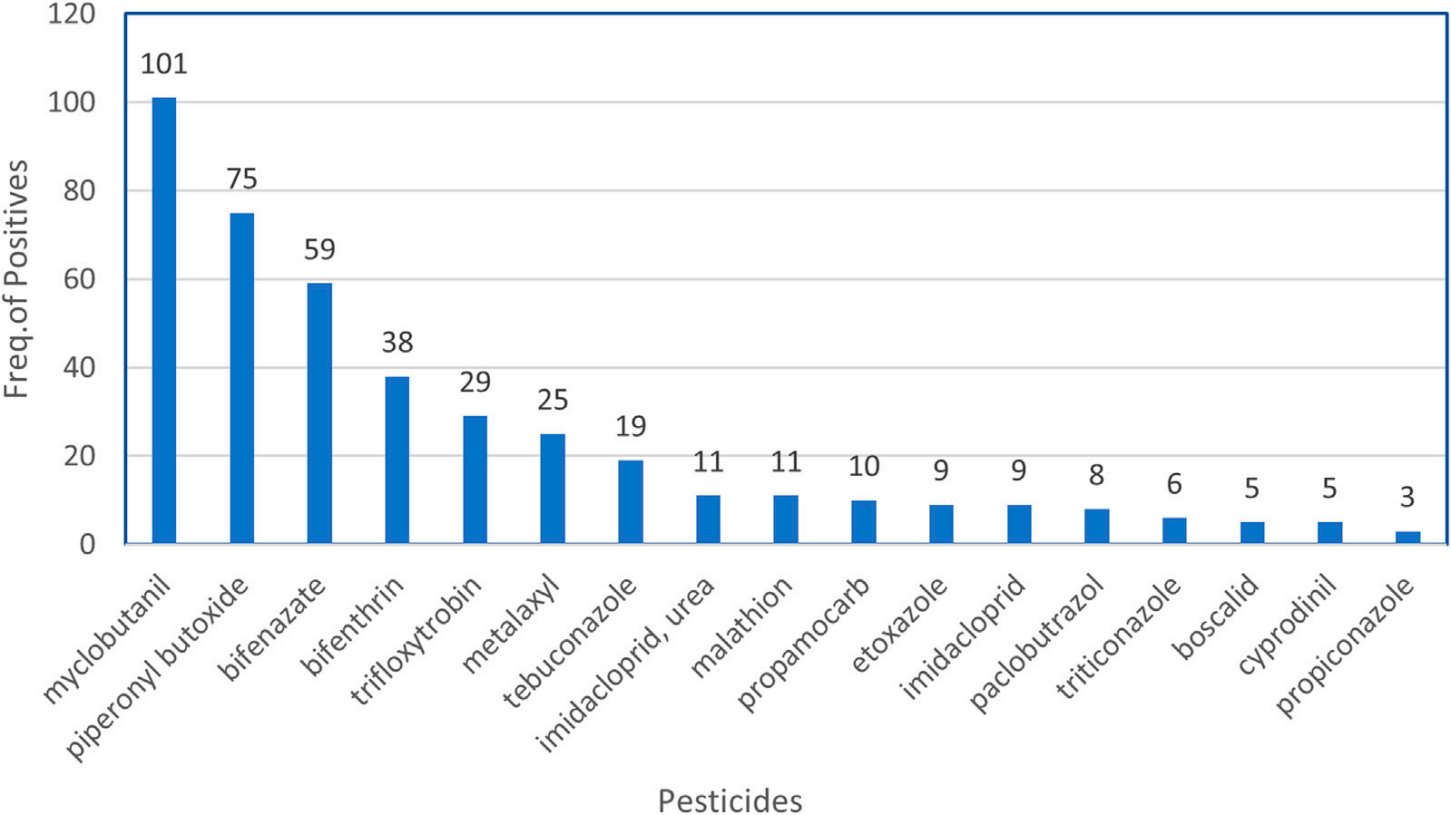
Frequently detected pesticides in illicit cannabis vaping fluids. At a detection level of >1 μg/g, the frequencies of the most commonly detected pesticides in the 202 cannabis vaping fluids are shown.

Unlike indoor cannabis cultivation that is highly regulated under the NYSMMP, black-market cannabis products may come from indoor or outdoor growing that utilizes pesticides. Low levels of pesticides and pesticide metabolites (>1 μg/g) were found in numerous cannabis and nicotine products obtained in this study. Of 74 nicotine vaping fluids tested, 12 (16%) were positive for either propamocarb (n = 11) or bentranil (n = 1). Of the 202 cannabis vaping fluids analyzed, 159 (79%) tested positive for various pesticides and pesticide metabolites, with individual samples containing up to 10 distinct pesticide residues. In total, 42 pesticides were detected in cannabis vaping fluids. The most detected pesticides are shown in Figure 5. Myclobutanil, a fungicide that is used to prevent the growth of powdery mildew on plants, was the most frequently detected, being present in 101 of 202 (50%) cannabis vaping fluids. The pesticide synergist, PBO, was detected in 75 (37%) of the samples. The pesticides bifenazate and bifenthrin were also frequently observed in cannabis vaping fluids. Aflatoxins B1, B2, G1, G2 or ochratoxin A were not detected in any of the vaping fluids analyzed in this study. Using our LC-HRMS/MS and GC-MS screening techniques together with our database searching methods, no synthetic cannabinoids, opiates, synthetic opioids, or other controlled substances were detected in any of the vaping fluids analyzed.

### Analysis of Commercial Cannabis Oil Diluents and Additives

At the onset of the NYSDOH investigation of EVALI in New York, shortly after our initial observation of VEA in EVALI case-associated vaping fluids, we questioned the source of VEA in the fluids. We noted that there were several commercial products of unknown composition marketed as “cannabis oil diluents” or “thickeners.” When these products were obtained and analyzed, several were found to be essentially pure VEA (Duffy et al., 2020). After a public health alert and subpoenas issued by the NYSDOH, these products are no longer available.

To investigate current or future chemicals that may be used in illicit vaping products, nine commercial “flavoring additives” were purchased from a commercial source. Untargeted GC-MS analysis was used to identify the components in the additives. From the results, it was apparent that most of the compounds in the flavoring additives were terpenes (Table 4). Caryophyllene was the most commonly detected terpene, which was present in eight of the nine product samples. Caryophyllene is a naturally occurring terpene in cannabis extracts. It can be used to adjust the flavor of a vaping fluid to better match that of a high-grade cannabis oil (Fournier et al., 1978; Gulluni et al., 2018; Ibrahim et al., 2019; Heblinski et al., 2020). Limonene was found in seven of the nine samples. d-Limonene is an aromatic terpene found in the citrus oils and it could possibly be used to produce a citrus flavor to the vaping fluids. However, limonene can be an irritant and have a bronchoconstrictive effect (Aronson 2015). No VEA was detected in these cannabis oil “flavoring additives.”

**TABLE 4:** Composition of commercial vaping-fluid additives.

Additive no	Components
1	citronellyl propionate; isophytol; *trans*-phytol, *cis*-phytol
2	exo-fenchol; α-terpineol, caryophyllene; α-humulene
3	Limonene; 1,2,3-Trimethylcyclopentene; α-Terpineol; caryophyllene; α-humulene
4	β-Myrcene; α-Terpinene; Limonene; terpinolene; isoborneol; endo-borneol; α-Terpineol; caryophyllene; α-humulene; α-Bisabolol
5	β-Pinene; Limonene; 4-Carene; isoborneol; α-Terpineol; caryophyllene; *cis*-β-ocimene; D-nerolidol
6	β-Myrcene; Limonene; β-linalool; caryophyllene
7	β-Myrcene; Limonene; γ-Terpinene; neomenthol; isoborneol; α-Terpineol; caryophyllene; α-Bisabolol
8	β-Myrcene; Limonene; γ-Terpinene; *cis*-Geraniol; caryophyllene; D-nerolidol
9	β-Myrcene; Limonene; γ-Terpinene; caryophyllene; α-humulene; D-nerolidol; α-Bisabolol

### Analysis of Nicotine Vaping Fluids

Many of the samples (82) that were submitted to our laboratory as part of our EVALI investigation were nicotine-containing pods. All of these appeared to be commercial products, and many of the samples had associated packaging and devices that supported their legitimacy as such. Nicotine vaping fluids were analyzed using untargeted GC-MS and LC-HRMS/MS. A summary of the major additives as estimated by GC-MS peak area is presented in Table 5. The e-liquids in nicotine-containing pods were found to contain the excipients, glycerin, propylene glycol and benzyl alcohol, along with various flavors (Margham et al., 2016). Benzoic acid, an additive to assist vaporization and improve absorption of nicotine salts, was detected in the nicotine products as has been in previous studies (Pankow et al., 2020). Benzoic acid is an acknowledged ingredient in e-liquids from Juul pods (Juul 2019). The cooling agents, WS-3 and WS-23, were also detected in nicotine-containing vaping fluids using GC-MS and confirmed using LC-HRMS/MS. These compounds provide a fast-acting cooling sensation and primarily affect the mouth and tongue (Behrendt et al., 2004; Sherkheli et al., 2010; Wang et al., 2014). When analyzed using GC-MS and LC-HRMS/MS, extracts of the nicotine pods that we received in association with EVALI cases did not reveal any chemical constituents that have not been previously reported other than trace pesticide residues.

**TABLE 5:** Major components found in nicotine-containing vaping fluids.

Additive	Benzoic acid
Carriers	Glycerin, Propylene glycol, Benzyl alcohol
Flavors	Triacetin, Vanillin, Levomenthol, menthol, Triethyl Citrate, Ethyl maltol
Cooling agents	WS-3, WS-23

## Conclusion

On September 5th, 2019, based on the initial observations from our laboratory, the NYSDOH announced an update on its investigation into vaping-associated pulmonary illness and issued the first in the nation public health advisory about VEA after high concentrations of VEA were found in the vaping devices recovered from EVALI patients (NYSDOH, 2019). In less than 2 years since that time, we have analyzed 284 samples from 83 cases of EVALI in New York State. While the overall number of cases of EVALI in New York State greatly declined over time, we observed remarkably similar rates of VEA positivity and levels of VEA content in case-associated vaping fluids as we did in the initial results leading to the health advisory. Whether the most recent samples containing VEA, *e.g*., received June 2021, represent remaining stock of illicit products that are slowly appearing on the black market or whether there is still limited use of VEA as a diluent is unknown. In this extended study, 132 (65%) of the cannabis vaping fluids recovered from EVALI patients contained VEA, and for EVALI cases in which one or more cannabis vaping product were submitted to the laboratory for analysis, 84% of the time at least one VEA-containing fluid was among the samples received. These results continue to support the initial hypothesis that VEA is causative in EVALI.

It should be noted that only the vaping fluid samples recovered at the time of diagnosis and hospitalization were analyzed in this study. While this may provide a snapshot of the patient’s chemical exposure, the fluids that were causally related to the onset of the condition may not have been submitted to the laboratory for analysis. Since the case-associated cannabis vaping products would have been illegal at the time of use, there may have been an under-submission of cannabis vaping products for analysis by the patients in favor of commercially available nicotine products (Ghinai et al., 2020). Despite these potential limitations, the association of EVALI with the use of VEA-containing cannabis vaping products is strong. The most convincing evidence for the role of VEA in EVALI came from the analyses of bronchoalveolar lavage fluids from EVALI patients. Vitamin E acetate was identified in BAL fluid obtained from 48 of 51 case patients (94%) from 16 states but not in such fluid obtained from the healthy comparator group (Blount et al., 2020).

How VEA may cause the condition of EVALI is not entirely clear; however, there are mechanistic studies that present several plausible mechanisms for VEA toxicity in vaping. At temperatures of 300 °C or higher, VEA undergoes pyrolysis and forms numerous toxic byproducts, including ketene (Wu and O'Shea, 2020) and duroquinone (Duffy et al., 2020). Ketene would be highly reactive with a variety of biomolecules. The duroquinone-durohydroquinone redox couple was observed in the vaping emissions from vitamin E acetate, which may be linked to acute oxidative stress and lung injuries (Jiang et al., 2020). While it is unclear whether inhalation of VEA causes lipoid pneumonia, it is known that vaporized VEA is an irritant to the lung mucosa and bronchi and can lead to chronic hypoxia (Cannon 1940). One or more of these mechanisms may lead to the EVALI condition. To date, the strongest evidence points to VEA as causative in EVALI.

This does not rule out potential harmful effects of other vaping components, such as replacement additives and diluents. For example, given the chemical properties of ricinoleic acid triglyceride, it could hardly be assumed that castor oil would be a safe component in the vaping scenario, as it could be expected to cause lipoid pneumonia (Cannon, 1940) and/or to generate reactive intermediates at high temperature. Aromatic/volatile hydrocarbons and oils consisting of MCT, terpenes and mineral oil in cannabis vaping fluids are suspected to cause oxidative stress and inflammatory responses in the lung (Chand et al., 2020). Recent studies in rats of phytol, one of the terpenes identified in commercial additives in this study, showed significant toxicity in respiratory tissue including dose-responsive tissue degeneration and necrosis in exposed animals that were in some instances associated with mortality (Schwotzer et al., 2021). These authors recommended that phytol not be used as an excipient in vaping products, as a safe exposure range for the compound has not been established. There is also no indication that long-term vaping of even low levels of pesticide residues in vaping fluids is without impact on pulmonary health. While the evidence that VEA is causative in EVALI is very strong, a decline in the use of VEA in the illicit cannabis oil market hardly means that the black-market vaping products are now safe, as some of the replacement additives and diluents also appear to elicit pulmonary toxicity.

## Data Availability

The datasets presented in this article are not readily available because; Data are associated with individual EVALI cases. Requests to access the datasets should be directed to DS, david.spink@health.ny.gov.
